# Curcumin treatment enhances bioactive metabolite accumulation and reduces enzymatic browning in soybean sprouts during storage

**DOI:** 10.1016/j.fochx.2023.100607

**Published:** 2023-02-21

**Authors:** Benliang Deng, Jing Zhao, Mengyao He, Shan Tian

**Affiliations:** a Life Science College, Luoyang Normal University, Luoyang 471934, Henan, China; b School of Life Science, Lanzhou University, Lanzhou 730000, Gansu, China; c Clinical Laboratory of Dingxi People’s Hospital, Dingxi City 743000, Gansu, China

**Keywords:** Enzymatic browning, H_2_O_2_ signaling, Polyphenol, Postharvest vegetable

## Abstract

Curcumin is a natural polyphenol that is widely used in food and medicine. Here, we investigated the effects of curcumin on the antioxidant accumulation and enzymatic browning of soybean sprouts after storage at 4 °C for 2 weeks. Curcumin drastically reduced the water loss, browning index, and peroxide accumulation, increased the activities of superoxide dismutase, catalase, and peroxidase, decreased the activities of phenylalanine ammonia-lyase and polyphenol oxidase, elevated the contents of ascorbic acid, reduced glutathione, nonprotein thiol, phenolics and isoflavones, and enhanced the total antioxidant capacity of soybean sprouts during storage. These curcumin-induced changes were partly but dramatically attenuated by inhibition of NADPH oxidase (NOX). Curcumin induced NOX activity and H_2_O_2_ burst in soybean sprouts during the first 24 h after treatment. The curcumin-induced antioxidants and -inhibited enzymatic browning are closely associated with NOX-dependent H_2_O_2_ signaling. The findings provide a new method for improving soybean sprout quality during storage.

## Highlights

• Curcumin inhibits enzymatic browning in soybean sprouts during storage.
• Curcumin affects the browning index, lipid damage, and antioxidant capacity.
• The effects of curcumin are largely reversed by DPI, an NOX inhibitor.
• The mechanism of action involves the activation of NOX-dependent H_2_O_2_ signaling.
• The findings provide a new method for maintaining vegetable quality during storage.

## 1 Introduction

Many elicitors induce antioxidant (e.g., polyphenol) accumulation in plants (Baenas, García-Viguera, & Moreno, 2014). Plants respond to elicitors by triggering an array of defense mechanisms including the production of reactive oxygen species (ROS) (Baenas et al., 2014). The ROS balance is tightly regulated by ROS generating [e.g., NADPH oxidase (NOX)] and scavenging systems, which consist of enzymatic antioxidants [e.g., superoxide dismutase (SOD) and catalase (CAT)] and non-enzymatic antioxidants including ascorbic acid and glutathione as well as polyphenols (Czarnocka & Karpiński, 2018). Interestingly, NOX has diverse ROS regulation functions in plants (Kaur, Sharma, Guruprasad, & Pati, 2014). For example, postharvest senescence in jujube fruit is delayed via NOX-mediated ROS signaling (Deng, Chen, Tian, Shi, & Zhao, 2022).

Curcumin, a natural polyphenolic compound obtained from the rhizomes of *Curcuma longa*, is a highly bioactive and coloring material (EI-Hack et al., 2021, Kocaadam and Şanlier, 2017). Curcumin has attracted much attention in food, medicine, and clinical industries (Esatbeyoglu et al., 2012, Kocaadam and Şanlier, 2017). Moreover, curcumin is a promising therapeutic agent due to its natural antioxidant properties and strong anti-inflammatory and antimicrobial functions (Esatbeyoglu et al., 2012). However, to our knowledge, no study has evaluated the potential functions of curcumin in regulating fruit and vegetable quality during storage.

Reducing postharvest losses of fruit and vegetables is critical due to the long distances between producers and consumers. Enzymatic browning, which is the second largest cause of quality loss in stored vegetables and fruit, has attracted considerable research attention (Gawlik-Dziki, Złotek, & Świeca, 2008). Polyphenol oxidase (PPO; EC 1.14.18.1) and peroxidase (POD; EC 1.11.1.7) participate in the rapid degradation of polyphenols and lead enzymatic browning in fruit and vegetables during storage (Sikora and Świeca, 2018, Tomás-Barberán and Espín, 2001). Phenylalanine ammonia-lyase (PAL; EC 4.3.1.1.) is a key enzyme in polyphenol biosynthesis and is also involved in enzymatic browning (Tomás-Barberán & Espín, 2001). Many methods have been developed to inhibit enzymatic browning (Qiao et al., 2021, Sikora and Świeca, 2018). However, no studies have revealed the potential roles of curcumin in modulating enzymatic browning in fruit and vegetables during storage.

Soybean sprouts contain abundant isoflavones and polyphenols (Chon, 2013, Le et al., 2019), which are required for enzymatic browning (Tomás-Barberán & Espín, 2001). In this study, we applied curcumin, a natural polyphenol, during storage of soybean sprouts to address the following two questions. First, does curcumin aggravate or alleviate enzymatic browning and antioxidant loss in soybean sprouts during storage? Second, does curcumin regulate the accumulation of bioactive (e.g., phenolics and isoflavones) nutrients and activities of browning-related enzymes in soybean sprouts during storage, and what are the potential mechanisms? This study is the first to investigate how curcumin regulates enzymatic browning and antioxidant nutrient accumulation in a stored vegetable.

## 2 Materials and methods

### 2.1 Reagent preparation

All the chemical reagents used here, including diphenyleneiodonium chloride (DPI), dimethyl sulfoxide (DMSO), and curcumin, were obtained from Macklin Biochemistry & Technique Company (Shanghai, China). For preparation of curcumin solution, 50 mg of curcumin was freshly dissolved in 1 mL of DMSO and then diluted with 100 mL of distilled water before filtering through a 0.22-μm membrane for sterilization. Ferric reducing ability of plasma (FRAP) solution was freshly prepared from 0.3 M sodium acetate buffer (pH 3.6), 0.02 M FeCl_3_, and 0.01 M 2,4,6-tripyridyl-s-triazine at a ratio of 10:1:1.

### 2.2 Experimental design

Soybean seeds (*Glycine* max L.) of Zhonghuang 37, a native cultivar provided by Shouguang Seed Co. (Weifang City, China), were used in this study. Seeds of identical size and weight were selected, surface sterilized in 1% (v/v) NaClO for 5 min, and then rinsed twice with distilled water before being steeped with distilled water at room temperature for 2 h. The seeds were then placed on trays lined with absorbent paper and kept in a dark chamber at room temperature and 90 % humidity for sprouting. The sprouts were harvested at day 5 after sowing. Before storage, the soybean sprouts were soaked with water (1% DMSO; as a control), curcumin, or curcumin + DPI (50 µM; DPI as an inhibitor of NOX was added to the curcumin solution before use) for 30 min. Subsequently, the treated soybean sprouts were manually collected, washed with distilled water, and kept at 4 °C and 70 % humidity under dark conditions. After treatment for 0, 6, 12, 18, and 24 h, the sprouts were sampled for the determination of NOX activity and H_2_O_2_ content. The sprouts stored for 0, 7, and 14 d were collected, rapidly frozen, and kept at −20 °C for subsequent analyses of weight loss, lipid damage, H_2_O_2_ content, antioxidant (ascorbic acid, glutathione, and nonprotein thiol) contents, accumulation and composition of phenolics and isoflavones, total antioxidant capacity, and enzyme (SOD, CAT, PAL, PPO, and POD) activities. For all assays, three replicates of 100 seeds were included for each treatment. All concentrations used in the current study were based on our preliminary study.

### 2.3 Evaluation of weight loss, enzymatic browning, lipid damage, and H_2_O_2_ content

Weight loss was evaluated by weighing each group of soybean samples before and after the storage period, and is presented as % weight loss compared with the initial weight. Enzymatic browning of soybean sprouts was measured at 420 nm using the method of Kim, Kim, Chung, and Moon (2014). Lipid damage was evaluated using the method of Dhindsa, Dhindsa, and Thorpe (1980) and the absorbance of thiobarbituric acid reactive substances (TBARS) was recorded at 532 and 600 nm. The H_2_O_2_ content was determined spectrophotometrically at 390 nm after reaction with KI (Velikova, Yordanov, & Edreva, 2000).

### 2.4 HPLC analysis of phenolic and isoflavone content

Soybean samples were prepared for analysis of phenolics and isoflavones by high-performance liquid chromatography (HPLC) by the method of Kim et al. (2006). Instrumentation for HPLC analysis was as described by Kim et al. (2006).

### 2.5 Determination of non-enzymatic antioxidant content and total antioxidant capacity

Ascorbic acid content was determined by 2,6-dichloro-phenol-indophenol titration (Ali, Khan, Malik, & Shahid, 2016). The reduced glutathione (GSH) and non-protein thiol content was measured by monitoring the absorbance at 412 nm with the method of Nagalakshmi and Prasad (2001). Total antioxidant capacity (evaluated by Fe^3+^ reducing power) was evaluated using the FRAP method (Benzie & Strain, 1996). The absorbance was recorded at 593 nm.

### 2.6 Enzyme activity assay

NOX activity was measured at 340 nm by the method of Grace and Logan (1996).

The activity of SOD (EC 1.15.1.1) was estimated at 560 nm based on the inhibition of nitroblue tetrazolium (NBT) reduction (Dhindsa et al., 1980).

The activity of CAT (EC 1.11.1.6) was estimated by monitoring the decrease in the absorbance of H_2_O_2_ at 240 nm (Dhindsa et al., 1980).

The activity of PAL was estimated using the method of Dickerson, Pascholati, Hagerman, Butler, and Nicholson (1984). One unit of PAL activity was defined as a change of 0.01 in absorbance at 290 nm per min at 25 °C.

The activity of PPO was estimated using the method of Gawlik-Dziki et al. (2008). One unit of PPO activity was defined as a change of 0.001 in absorbance at 420 nm per min at 25 °C.

The activity of POD was estimated at 470 nm using the method of Khalil, Yusuf, Bassuony, Gamal, and Madany (2020). POD activity was expressed as mol kg^−1^ min^−1^.

The activities of enzymes (NOX, SOD, CAT, PAL, PPO, and POD) were measured spectrophotometrically and expressed on a protein basis. The protein concentration of the extract was determined using the method of Bradford (1976).

### 2.7 Data analysis

All experiments were carried out in triplicate with a completely randomized design. All data analysis was done using Duncan’s multiple range test using SPSS 20 statistical software (IBM Corp., Armonk, NY, USA). The significance level was *p* ≤ 0.05.

## 3 Results

### 3.1 Curcumin enhances the antioxidant capacity of soybean sprouts

Compared with before storage (0 d), the total antioxidant capacity, ascorbic acid content, GSH content, and nonprotein thiol content of the soybean sprouts decreased by approximately 45 %, 75 %, 81 %, and 49 %, respectively, after storage for 14 d at 4 °C (Table 1; *p* < 0.05). Curcumin treatment increased the total antioxidant capacity, ascorbic acid content, GSH content, and nonprotein thiol content after storage for 14 d by approximately 52 %, 168 %, 191 %, and 72 %, respectively (Table 1; *p* < 0.05). However, after treatment with both curcumin and DPI (curcumin + DPI), the total antioxidant capacity, ascorbic acid content, GSH content, and nonprotein thiol content were decreased by approximately 25 %, 43 %, 47 %, and 30 %, respectively, compared with the soybean sprouts treated with curcumin only after storage for 14 d (Table 1; *p* < 0.05).

**Table 1:** Curcumin affects antioxidants in soybean sprouts during storage.

	Day 0	Day 14		
	Control	Control	Curcumin	+ DPI
TAC (mol kg^−1^)	4.84 ± 0.15^a^	2.67 ± 0.14^d^	4.07 ± 0.29^b^	3.06 ± 0.11^c^
AA (mmol kg^−1^)	0.75 ± 0.05^a^	0.19 ± 0.04^d^	0.51 ± 0.06^b^	0.29 ± 0.04^c^
GSH (mmol kg^−1^)	0.59 ± 0.03^a^	0.11 ± 0.03^d^	0.32 ± 0.02^b^	0.17 ± 0.03^c^
NPT (mmol kg^−1^)	6.21 ± 0.42^a^	3.18 ± 0.52^d^	5.48 ± 0.48^b^	3.81 ± 0.23^c^

Curcumin regulates total antioxidant capacity (TAC), ascorbic acid (AA) content, reduced glutathione (GSH) content, and nonprotein thiol content (NPT) of soybean sprouts during storage at 4 °C. Means associated with the same letter are not significantly different (*n* = 3; *p* < 0.05). DPI, diphenyleneiodonium chloride.

### 3.2 Curcumin enhances the phenolic and isoflavone content of soybean sprouts

Compared with before storage (0 d), the total phenolic content of the soybean sprouts, and of the major components *p*-coumaric acid, ferulic acid, naringin, hesperidin, and salicylic acid, decreased by approximately 37 %, 47 %, 30 %, 27 %, 43 %, and 68 %, respectively, after storage for 14 d at 4 °C (Table 2; *p* < 0.05). Curcumin treatment increased the total phenolic content and the content of *p*-coumaric acid, ferulic acid, naringin, hesperidin, and salicylic acid, after storage for 14 d by approximately 41 %, 50 %, 27 %, 26 %, 52 %, and 175 %, respectively, over the control (Table 2; *p* < 0.05). However, after treatment with both curcumin and DPI (curcumin + DPI), the total content of phenolics and of *p*-coumaric acid, ferulic acid, naringin, hesperidin and salicylic acid decreased by approximately 15 %, 25 %, 8 %, 13 %, 19 %, and 45 %, respectively, compared with the soybean sprouts treated with curcumin only, after storage for 14 d (Table 2; *p* < 0.05).

**Table 2:** Curcumin affects the content of phenolics and isoflavones and their composition in soybean sprouts during storage.

	Day 0	Day 14		
	Control	Control	Curcumin	+ DPI
TPC (mg kg^−1^)	30.9 ± 1.3^a^	19.4 ± 1.6^d^	27.4 ± 1.1^b^	23.2 ± 1.3^c^
*p*-Coumaric acid	1.5 ± 0.1^a^	0.8 ± 0.1^c^	1.2 ± 0.1^b^	0.9 ± 0.2^b^
Ferulic acid	17.6 ± 0.5^a^	12.4 ± 1.1^c^	15.8 ± 0.8^b^	14.6 ± 0.6^b^
Naringin	2.6 ± 0.2^a^	1.9 ± 0.2^b^	2.4 ± 0.1^a^	2.1 ± 0.1^b^
Hesperidin	5.4 ± 0.2^a^	3.1 ± 0.3^d^	4.7 ± 0.3^b^	3.8 ± 0.2^c^
Salicylic acid	3.8 ± 0.2^a^	1.2 ± 0.1^d^	3.3 ± 0.2^b^	1.8 ± 0.1^c^
TFC (mg kg^−1^)	589.2 ± 18.2^a^	224.7 ± 21.2^d^	494.8 ± 15.2^b^	328.1 ± 13^c^
Daidzin	193.8 ± 13.1^a^	63.3 ± 8.8^d^	167.2 ± 11.3^b^	106.1 ± 10.4^c^
Glycitin	31.4 ± 1.9^a^	14.2 ± 0.9^d^	25.1 ± 1.7^b^	19.3 ± 1.7^c^
Genistin	274.6 ± 12.7^a^	112.1 ± 15.1^d^	229.3 ± 16.5^b^	157.2 ± 11.4^c^
Daidzein	70.8 ± 4.7^a^	25.9 ± 2.2^d^	58.1 ± 4.2^b^	34.2 ± 3.1^c^
Genistein	18.2 ± 1.7^a^	9.2 ± 0.8^d^	15.1 ± 0.9^b^	11.3 ± 0.7^c^

Curcumin regulates the total content of phenolics (TPC) and isoflavones (TFC), and the major components of these secondary metabolites of soybean sprouts, during storage at 4 °C. Means associated with the same letter are not significantly different (*n* = 3; *p* < 0.05). DPI, diphenyleneiodonium chloride.

Similar patterns of change were also determined for isoflavone accumulation (Table 2). Compared with before storage (0 d), the total isoflavone content decreased by approximately 62 %, 16 %, and 44 % in water-, curcumin-, and curcumin + DPI-treated soybean sprouts after storage for 14 d (Table 2; *p* < 0.05). Treatment with curcumin increased the content of daidzin, glycitin, genistin, daidzein, and genistein in soybean sprouts by approximately 164 %, 77 %, 105 %, 124 %, and 64 %, respectively, compared with controls, after storage for 14 d (Table 2; *p* < 0.05). However, after treatment with both curcumin and DPI (curcumin + DPI), the daidzin, glycitin, genistin, daidzein, and genistein contents were decreased by approximately 63 %, 23 %, 31 %, 41 %, and 25 %, respectively, compared with soybean sprouts treated with curcumin only, after storage for 14 d (Table 2; *p* < 0.05).

### 3.3 Curcumin reduces postharvest quality loss and enhances peroxide accumulation

**Fig. 1:**
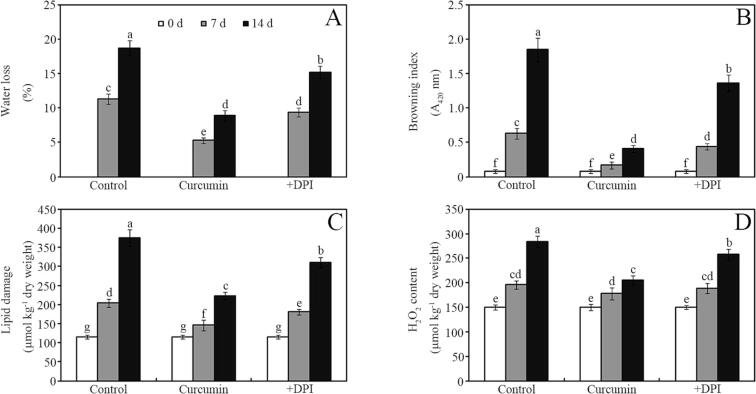
Curcumin reduces browning and lipid peroxidation. Curcumin regulates the water loss (A), browning index (B), lipid peroxidation (C), and H_2_O_2_ content (D) of soybean sprouts during storage at 4 °C. Bars represent standard deviation of the mean (*n* = 3); means associated with the same letter are not significantly different (*p* < 0.05). DPI, diphenyleneiodonium chloride.

Curcumin reduced water loss and enzymatic browning in the soybean sprouts. However, this curcumin-induced reduction in water loss and enzymatic browning was attenuated by treatment with 50 µM DPI, a specific inhibitor of NOX (Fig. 1a and b). Treatment with curcumin decreased the water loss and browning index of soybean sprouts by approximately 52 % and 78 %, respectively, after storage for 14 d (Fig. 1b; *p* < 0.05). However, the water loss and browning indexes of soybean sprouts treated with curcumin + DPI were approximately 71 % and 232 % higher, respectively, after storage for 14 d compared with those of the sprouts treated with curcumin only (Fig. 1b; *p* < 0.05).

Curcumin decreased lipid damage (evaluated based on the TBARS content) and H_2_O_2_ content in soybean sprouts during storage (Fig. 1c; *p* < 0.05). For example, curcumin treatment decreased the TBARS and H_2_O_2_ content of soybean sprouts by approximately by 41 % and 27 %, respectively, after storage for 14 d (Fig. 1c). This curcumin-induced decrease in TBARS and H_2_O_2_ content was reversed by DPI. For example, the TBARS and H_2_O_2_ contents of sprouts treated with curcumin + DPI were approximately 24 % and 6 % higher, respectively, compared with those of sprouts treated with curcumin only after storage for 7 d (Fig. 1c; *p* < 0.05).

### 3.4 Curcumin increases NOX activity and H_2_O_2_ content

**Fig. 2:**
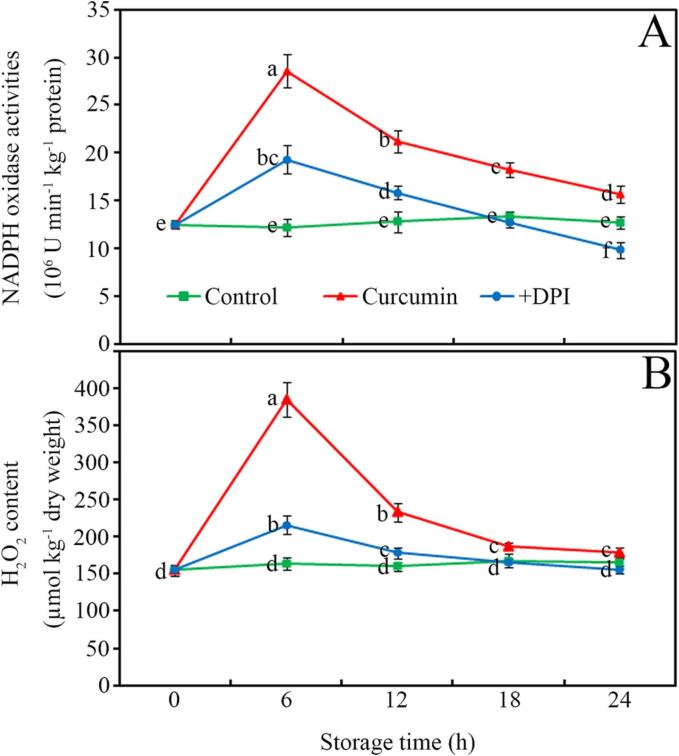
Curcumin enhances NADPH oxidase activity and H_2_O_2_ content. Curcumin regulates the NADPH oxidase activity (A) and H_2_O_2_ content (B) of stored soybean sprouts after treatment for 0, 6, 12, 18, and 24 h. Bars represent standard deviation of the mean (*n* = 3); means associated with the same letter are not significantly different (*p* < 0.05). DPI, diphenyleneiodonium chloride.

Curcumin treatment increased the NOX activity and induced H_2_O_2_ burst in soybean sprouts within 24 h (Fig. 2). For example, curcumin treatment increased the NOX activity by approximately 134 %, 66 %, 37 %, and 24 % in soybean sprouts stored for 6, 12, 18, and 24 h, respectively (Fig. 2a; *p* < 0.05). However, the NOX activities of the sprouts treated with curcumin + DPI were approximately 33 %, 25 %, 30 %, and 38 % lower than those of the sprouts treated with curcumin alone after storage for 6, 12, 18, and 24 h, respectively (Fig. 2a; *p* < 0.05). Similar trends were observed in H_2_O_2_ content (Fig. 2b). For example, treatment with curcumin and curcumin + DPI increased the H_2_O_2_ content of soybean sprout by approximately 135 % and 32 %, respectively, after storage for 6 h (Fig. 2b; *p* < 0.05).

### 3.5 Curcumin modulates enzyme activities

**Fig. 3:**
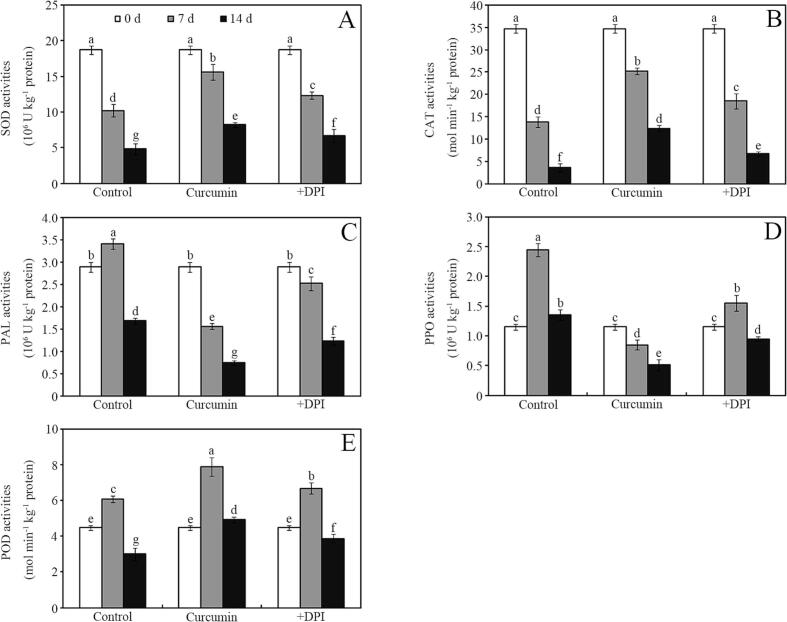
Curcumin affects the enzyme activities. Curcumin regulates the activities of SOD (A), CAT (B), PAL (C), PPO (D), and POD (E) in soybean sprouts during storage at 4 °C. Bars represent standard deviation of the mean (*n* = 3); means associated with the same letter are not significantly different (*p* < 0.05). SOD, superoxide dismutase; CAT, catalase; PAL, phenylalanine ammonia-lyase; PPO, polyphenol oxidase; POD, peroxidase; DPI, diphenyleneiodonium chloride.

Curcumin treatment enhanced the SOD and CAT activities in stored soybean sprouts compared with the control (Fig. 3a,b). However, these curcumin-induced increases in SOD and CAT activities were reversed by the addition of 50 µM DPI (Fig. 3a,b; *p* < 0.05). For example, compared with the control, treatments with curcumin and curcumin + DPI increased the SOD activity of soybean sprout by approximately 71 % and 40 %, respectively, after storage for 14 d (Fig. 3a; *p* < 0.05). Similarly, curcumin treatment increased the CAT activity by approximately 83 % and 244 % after storage for 7 and 14 d, respectively, compared with the control (Fig. 3b; *p* < 0.05). Meanwhile, compared with the CAT activities of sprouts treated with curcumin alone, those of sprouts treated with curcumin + DPI were approximately 27 % and 46 % lower after storage for 7 and 14 d, respectively (Fig. 3b; *p* < 0.05).

Curcumin affected the activities of PAL, PPO, and POD in stored soybean sprouts (Fig. 3c-e), and these effects were reversed by the addition of 50 µM DPI (Fig. 3c-e). After storage for 14 d, curcumin reduced the PAL and PPO activities by approximately 74 % and 56 %, respectively, compared with the control (Fig. 3c,d; *p* < 0.05). After storage for 7 d, the PAL and PPO activities of the sprouts treated by curcumin + DPI were approximately 62 % and 82 % higher than those of the sprouts treated with curcumin only (Fig. 3c,d; *p* < 0.05). Treatments with curcumin and curcumin + DPI enhanced the POD activity of soybean sprouts stored for 14 d by approximately 64 % and 29 %, respectively, compared with the control (Fig. 3e; *p* < 0.05).

## 4 Discussion

In this study, curcumin reduced the water loss, enzymatic browning, lipid damage and H_2_O_2_ accumulation in stored soybean sprouts compared with controls (Fig. 1). Curcumin can simultaneously alleviate enzymatic browning and lipid damage in fruit and vegetables after harvest (Pasquariello et al., 2015). In the present study, the curcumin-induced suppression of water loss, enzymatic browning, lipid damage, and H_2_O_2_ content was attenuated by DPI, a specific inhibitor of NOX (Ge et al., 2015), which is a major ROS-producing enzyme (Kaur et al., 2014). This indicates that the curcumin-induced suppression of postharvest vegetable quality decline (water loss, enzymatic browning, lipid damage, and H_2_O_2_ content) is closely associated with NOX-dependent H_2_O_2_ production. Exogenous H_2_O_2_ has been shown to drastically inhibit the browning of fresh-cut fruit (Chumyam et al., 2019, Peng et al., 2008).

In the present study, curcumin enhanced NOX activity and H_2_O_2_ burst in stored soybean sprouts within 24 h after treatment (Fig. 2). However, these effects of curcumin were reversed by DPI (Fig. 2). NOX-mediated ROS production in *Botrytis cinerea* is required for curcumin to inhibit pathogen growth in the plant (Hua et al., 2019), and NOX-mediated H_2_O_2_ signaling has been shown to trigger the antioxidant systems of plants (Jiang and Zhang, 2003, Kaur et al., 2014). Interestingly, the pro-oxidant activity of polyphenol was found to enhance antioxidant capacity in human cells (Tedesco et al., 2021). Moreover, antioxidants such as CAT, ascorbic acid, and GSH can efficiently suppress enzymatic browning in fruit and vegetables after harvest (Qiao et al., 2021, Sikora and Świeca, 2018, Wu, 2014). Thus, the curcumin-induced inhibition of enzymatic browning may be attributed to its own ability to enhance antioxidant capacity, leading us to question whether curcumin improved the antioxidant capacity of stored soybean sprouts.

To test the above idea, we investigated the effects of curcumin on the activities of antioxidant enzymes (e.g., SOD and CAT) and non-enzymatic antioxidants (e.g., ascorbic acid and GSH) in soybean sprouts. As shown in Fig. 3a,b, curcumin enhanced the SOD and CAT activities in stored soybean sprouts compared with the control. This finding is partly in accordance with Hua et al. (2019), who reported that curcumin increased the activities of antioxidant enzymes in postharvest kiwifruit. Similarly, in our study, curcumin increased the total antioxidant capacity, ascorbic acid content, GSH content, nonprotein thiol accumulation, and total phenolic and isoflavone content, in soybean sprouts compared with controls (Table 1, Table 2). How does curcumin improve antioxidant capacity? One plausible explanation may be related to the pro-oxidant activity of curcumin in the presence of transition metal ions like Fe^2+^ and Cu^2+^ (Yoshino et al., 2004), which may be released by senescent cells of fruit and vegetables during storage. However, the curcumin-induced enhancement in antioxidant capacity was reversed by DPI (Fig. 3a,b and Table 1, Table 2), an inhibitor of NOX. Thus, the effect of curcumin on antioxidant capacity was closely associated with NOX-dependent H_2_O_2_ signaling.

PPO and POD, which are related to polyphenol degradation, along with PAL are thought to be the main enzymes responsible for enzymatic browning (Tomás-Barberán & Espín, 2001). Ascorbic acid and GSH have been shown to suppress the activity of PPO (Gacche et al., 2004, Landi et al., 2013). Thus, we investigated whether curcumin regulates the activities of these browning-related enzymes in stored soybean sprouts. As shown in Fig. 3c-e, curcumin reduced the activities of PAL and PPO but enhanced the activity of POD in soybean sprouts compared with the control. Interestingly, curcumin was found to profoundly inhibit the activities of PAL (Jhin & Hwang, 2015) and PPO (Aksoy, 2020) *in vitro*. This suggests that the curcumin-induced reduction in enzymatic browning can be partly attributed to the inhibition of the activities of PAL and PPO in stored soybean sprouts. However, the effects of curcumin on the activities of browning-related enzymes (PAL, PPO, and POD) were reversed by DPI (Fig. 3c-e). Thus, the PAL, PPO, and POD activities were also affected by the oxidative status, which can be regulated by curcumin in soybean sprouts during storage. Moreover, phenolics are located in vacuoles, while PPO and POD are located in plastids and other organelles (Jiang, Duan, Joyce, Zhang, & Li, 2004). This cellular compartmentalization can prevent PPO and POD from contacting phenolic substrates, effectively avoiding enzymatic browning in normal fruit and vegetables (Jiang et al., 2004). In our study, curcumin reduced lipid damage and maintained the integrity of the cellular membrane structure by increasing the ROS scavenging capacity in soybean sprouts (Table 1; Fig. 1b; Fig. 3a,b). Thus, curcumin deterred cellular decompartmentalization and prevented PPO and POD from contacting phenolics to form brown polymers (Jiang et al., 2004).

**Fig. 4:**
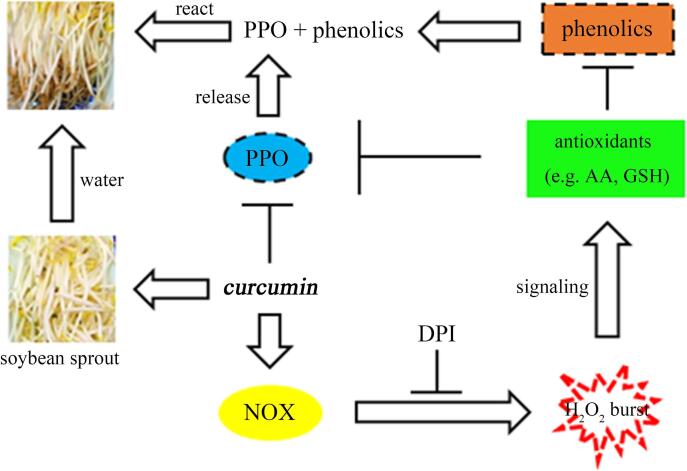
Hypothetical model for how curcumin inhibits enzymatic browning. A hypothetical model for curcumin-induced inhibition of enzymatic browning in soybean sprouts during storage via activation of NOX-mediated H_2_O_2_ signaling. The sharp and blunt arrows represent positive and negative effects, respectively. Curcumin enhanced the antioxidant capacity, which inhibited the activity of PPO, and prevented contact between phenolics and PPO by maintaining the membrane integrity. For more details, see the text. AA, ascorbic acid; NOX, NADPH oxidase; PPO, polyphenol oxidase; DPI, diphenyleneiodonium chloride.

For a better understanding, we developed a hypothetical model based on NOX-mediated H_2_O_2_ signaling to illustrate how curcumin hinders antioxidant loss and reduces enzymatic browning in stored soybean sprouts (Fig. 4). In this model, curcumin triggers NOX-mediated H_2_O_2_ signaling, which activates the antioxidant system in stored soybean sprouts. The antioxidants inhibit the activity of PPO, a key enzyme for enzymatic browning. Moreover, the enhanced antioxidant capacity helps maintain the membrane integrity, which prevents phenolics from contacting PPO. Based on the current study and published data, this model provides a new pathway through which curcumin inhibits enzymatic browning in soybean sprouts during storage. However, additional experimental evidence is required to clarify the detailed mechanism.

## 5 Conclusions

Some interesting conclusions can be drawn from this study. First, curcumin inhibited water loss, enzymatic browning, lipid peroxidation, and H_2_O_2_ content, and decreased the activities of PPO and PAL in soybean sprouts during storage. Second, curcumin triggered NOX-dependent H_2_O_2_ signaling to inhibit enzymatic browning. Third, curcumin enhanced the activities of antioxidant enzymes (e.g., SOD and CAT) and reduced the loss of antioxidants (e.g., ascorbic acid and GSH) in soybean sprouts during storage. This is the first study to demonstrate that curcumin suppresses enzymatic browning in stored vegetables through NOX-mediated H_2_O_2_ signaling. The findings provide evidence for the application of curcumin to preserve soybean sprouts.

## Funding

This work was supported by the “Introduced Talent Person of Luoyang Normal University” to Benliang Deng (Grant number: 190141051001).

## CRediT authorship contribution statement

**Benliang Deng:** Conceptualization, Investigation, Supervision. **Jing Zhao:** Investigation, Data curation, Methodology. **Mengyao He:** Investigation, Data curation, Methodology. **Shan Tian:** Investigation, Data curation, Methodology.

## Declaration of Competing Interest

The authors declare that they have no known competing financial interests or personal relationships that could have appeared to influence the work reported in this paper.

## Data Availability

Data will be made available on request.
